# Supplementary material for: Ethanol Exposure Increases Oxygen Consumption by Developing Cerebral Arteries in a Trimester-, Concentration- and Sex-Dependent Manner
Source: Biomolecules. 2025 Nov 7;15(11):1566. doi: 10.3390/biom15111566 (PMC12650377; doi:10.3390/biom15111566)
Supplement: Supplementary file 1 [file biomolecules-15-01566-s001.zip › biomolecules-3944458-supplementary.pdf]

SUPPLEMENTARY FIGURE AND TABLE LEGENDS

Supplementary Figure S1. Schematic of Seahorse XF Cell Mito Stress Test assay and analysis of mitochondrial respiration parameters. **(A)** Schematic representation of the mitochondrial electron transport chain showing the specific sites targeted by different drugs used in the in Mitochondrial Stress Test: oligomycin inhibits ATP synthase (Complex V), FCCP acts as a protonophore to uncouple oxidative phosphorylation, while rotenone and antimycin A inhibit Complex I and Complex III, respectively. **(B)** Representative trace of oxygen consumption rate (OCR) illustrates the order of drugs being probed and the resulting bioenergetic parameters: basal respiration, ATP production, proton leak, maximal respiration, non-mitochondrial respiration, spare respiratory capacity, and coupling efficiency. **(C)** Representative trace of OCR highlighting the analysis approach used where non-mitochondrial respiration rate was subtracted to obtain mitochondria-specific OCR values.

Supplementary Figure S2. Validation of sex separation in third trimester equivalent pups. **(A)** Representative images of male (left) and female (right) pups, with female sex identified by the apparent presence of mammary gland nipples (red arrows). **(B)** Sex identification was further confirmed by qPCR analysis of the *Sry* gene transcript in liver tissue samples. In the bar graph, the y-axis represents $2^{-\Delta Ct}$, where Ct indicates the cycle number at which the fluorescence threshold is crossed to detect the target gene. *Sry* gene product in each sample was normalized to *Gapdh* coding transcript. Each data point represents an individual pup (male n = 8, female n = 7); bars indicate mean ± SEM. *$p < 0.05$, unpaired 2-tailed t-test.

Supplementary Figure S3. Blood ethanol levels in dams and pups measured at the same timepoint. **(A)** Maternal blood ethanol levels measured 2 hours after first ethanol exposure (6 g/kg). Each point represents an individual dam (n = 4). **(B)** Pup blood ethanol levels measured 2 hours after maternal ethanol exposure (6 g/kg). Each point represents data collected from a single pup (n = 14). Data are shown as mean ± SEM.

Supplementary Table S1. Distribution of fetuses across litters following ethanol exposure during GD 9-19 (second trimester equivalent paradigm of maternal ethanol exposure). Table shows the number of offsprings per litter across experimental groups.

Supplementary Table S2. Distribution of male and female pups across litters following ethanol exposure during PD 1-10 (third trimester equivalent paradigm of maternal ethanol exposure). Table shows the number of male and female offspring per litter across experimental groups. Each litter was separated for sex initially by appearance of mammary glands. Sex separation was subsequently confirmed by qPCR detection of the *Sry* gene transcript in males.

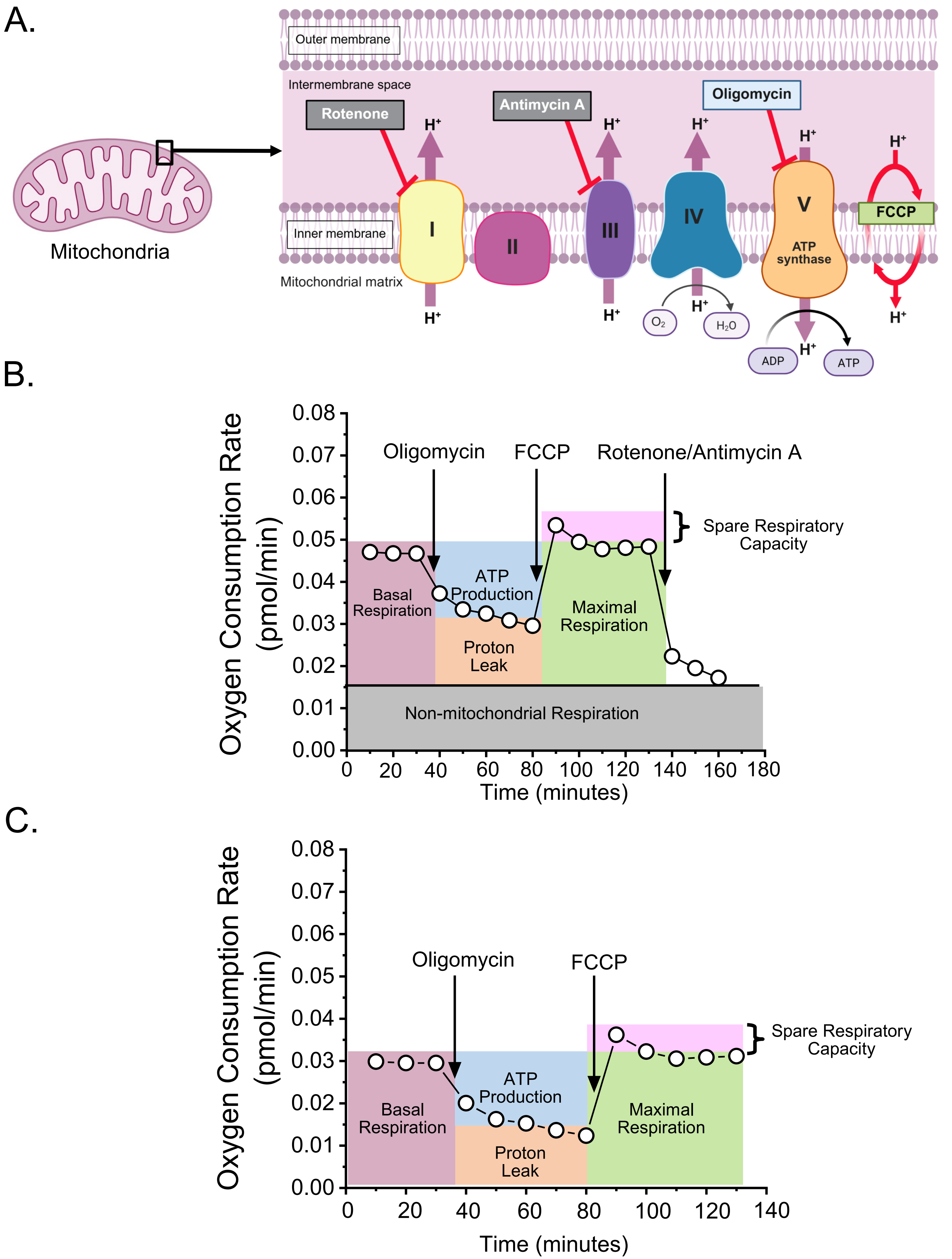

Supplementary Figure S1.

A.

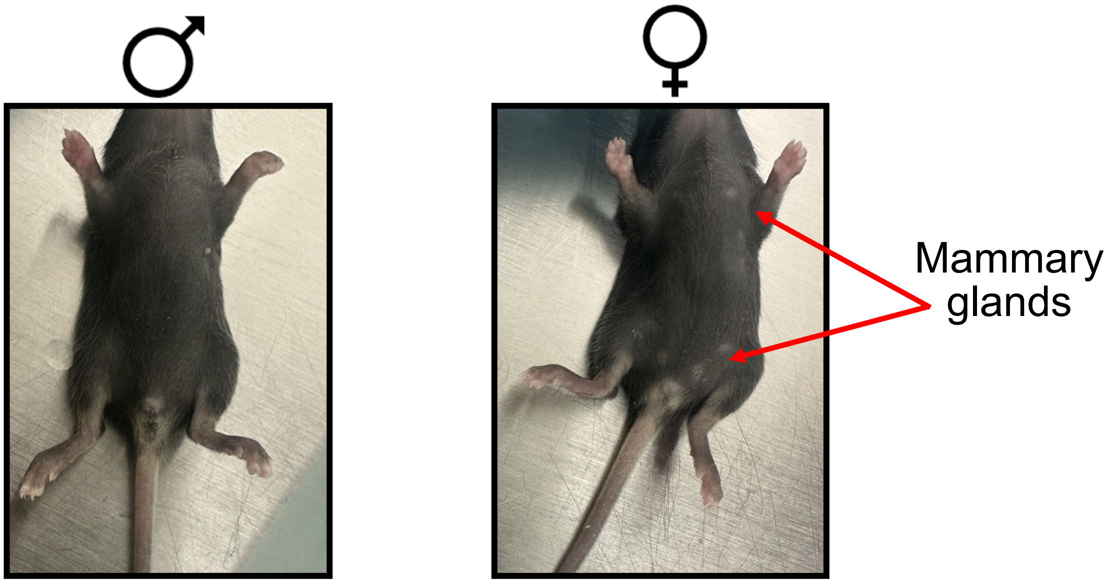

B.

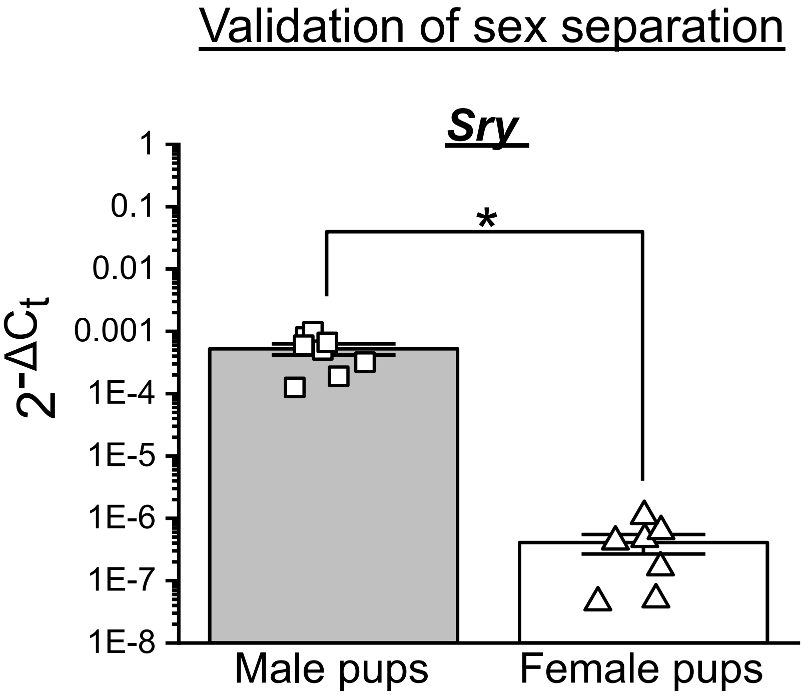

Supplementary Figure S2.

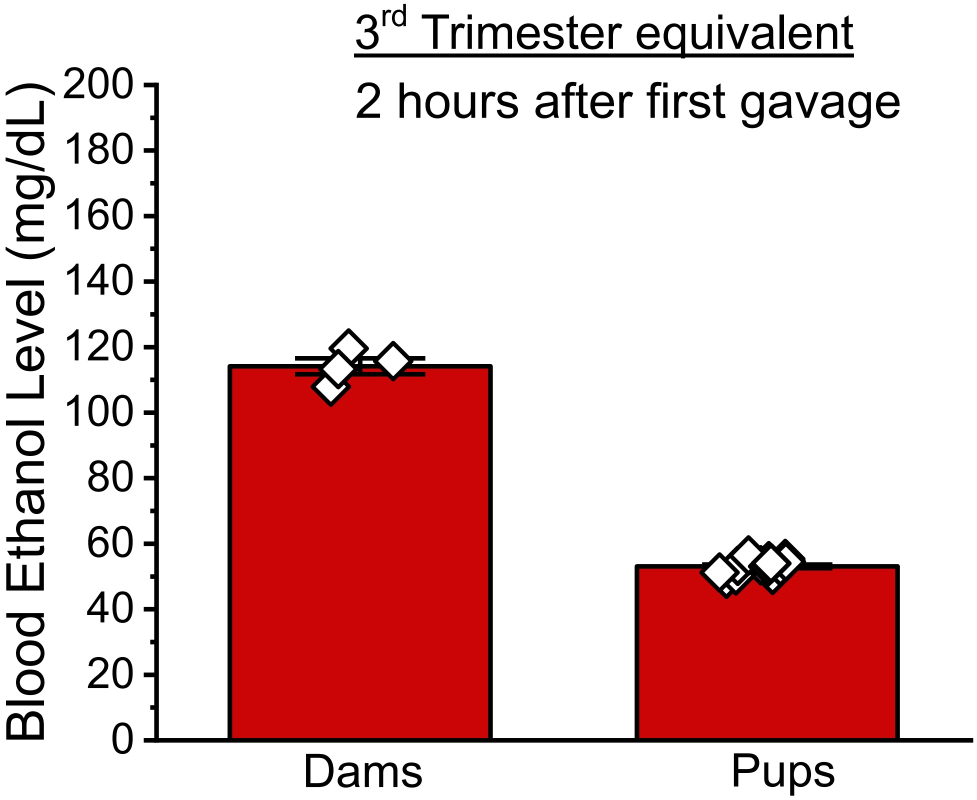

Supplementary Figure S3.

2nd Trimester equivalent

	Litter ID	# of fetuses		Litter ID	# of fetuses
CONTROL GROUP	Set 11 F1	9	ALCOHOL EXPOSED GROUP	Set 11 F2	7
	Set 11 F4	11		Set 11 F3	10
	Set 11 F5	9		Set 11 F6	8
	Set 14 F2	8		Set 14 F1	9
	Set 14 F4	8		Set 14 F3	8
	Set 14 F5	9		Set 15 F2	8
	Set 15 F1	8		Set 15 F3	6
	Set 15 F7	11		Set 15 F5	7
	Set 15 F8	7		Set 20 F1	6
	Set 20 F6	6		Set 20 F2	6
	Set 20 F8	7		Set 22 F1	7
	Set 22 F8	6		Set 22 F3	6
	Set 26 F3	10		Set 26 F2	7
	Set 26 F4	9		Set 27 F1	6
	Set 26 F7	9		Set 27 F4	9
	Set 27 F2	8		Set 29 F2	9
	Set 27 F5	8		Set 29 F3	9
	Set 27 F10	6		Set 29 F4	8
	Set 29 F1	8		Set 29 F8	9
	**Total #**	157		**Total #**	145

Supplementary Table S1

	Litter ID	# of ♂	# of ♀		Litter ID	# of ♂	# of ♀
CONTROL GROUP	Set 21 F1	2	2	ALCOHOL EXPOSED GROUP	Set 21 F3	5	4
	Set 21 F2	3	3		Set 21 F4	4	5
	Set 21 F5	3	5		Set 21 F8	2	5
	Set 21 F14	4	5		Set 21 F13	4	0
	Set 24 F9	2	2		Set 24 F1	4	1
	Set 24 F11	4	5		Set 24 F3	3	3
	Set 25 F1	3	4		Set 24 F4	3	2
	Set 25 F9	3	1		Set 24 F6	1	5
	Set 25 F11	4	4		Set 24 F8	4	3
	Set 30 F4	3	4		Set 25 F3	4	4
	Set 30 F8	3	1		Set 25 F4	3	4
	Set 30 F14	6	0		Set 25 F10	6	2
	Set 31 F9	3	4		Set 25 F12	3	0
	Set 33 F4	4	4		Set 30 F1	4	3
	Set 33 F11	3	2		Set 30 F2	7	1
	Set 33 F13	1	2		Set 30 F7	6	4
	Set 35 F2	0	5		Set 30 F11	4	5
	Set 35 F4	3	3		Set 31 F1	0	8
	Set 35 F7	2	3		Set 31 F3	3	5
	Set 35 F8	3	3		Set 31 F5	5	2
	Set 35 F17	4	2		Set 31 F7	8	0
					Set 31 F10	5	3
					Set 31 F13	3	5
					Set 33 F1	6	0
					Set 33 F3	4	1
					Set 33 F6	4	3
					Set 33 14	3	3
					Set 35 F6	3	4
					Set 35 F10	3	4
					Set 35 F13	3	1
					Set 35 F15	3	3
					Set 35 F19	5	3
					Set 35 F20	3	3
	**Total #**	63	64		**Total #**	128	99

Supplementary Table S2
